# Impact of the COVID-19 pandemic on mental health among local residents in South of Brazil: during pandemic times, youth sleep matters

**DOI:** 10.47626/2237-6089-2021-0225

**Published:** 2021-11-09

**Authors:** Joana Bücker, Adriane Ribeiro Rosa, Letícia Sanguinetti Czepielewski

**Affiliations:** 1 Programa de Pós-Graduação em Ciências Médicas Universidade do Vale do Taquari Lajeado RS Brazil Programa de Pós-Graduação em Ciências Médicas, Universidade do Vale do Taquari (UNIVATES), Lajeado, RS, Brazil.; 2 Laboratório de Psiquiatria Molecular Hospital de Clínicas de Porto Alegre Porto Alegre RS Brazil Laboratório de Psiquiatria Molecular, Hospital de Clínicas de Porto Alegre (HCPA), Porto Alegre, RS, Brazil.; 3 Departamento de Farmacologia Universidade Federal do Rio Grande do Sul Porto Alegre RS Brazil Departamento de Farmacologia, Universidade Federal do Rio Grande do Sul (UFRGS), Porto Alegre, RS, Brazil.; 4 Programa de Pós-Graduação em Psiquiatria e Ciências do Comportamento UFRGS Porto Alegre RS Brazil Programa de Pós-Graduação em Psiquiatria e Ciências do Comportamento, UFRGS, Porto Alegre, RS, Brazil.; 5 Departamento de Psicologia do Desenvolvimento e da Personalidade Instituto de Psicologia UFRGS Porto Alegre RS Brazil Programa de Pós-Graduação em Psicologia, Departamento de Psicologia do Desenvolvimento e da Personalidade, Instituto de Psicologia, UFRGS, Porto Alegre, RS, Brazil.

**Keywords:** COVID-19, mental health, sleep quality

## Abstract

**Introduction:**

Social isolation has been associated with poor sleep quality and mental health problems during the COVID-19 pandemic. However, most studies have investigated heterogeneous samples subjected to varying social distancing policies and did not focus on a single local profile subject to homogeneous prevention policies.

**Objective:**

To evaluate the impact of the COVID-19 pandemic on mental health and sleep quality in a specific region in the South of Brazil where the populations have similar culture and local governments have adopted similar social distancing policies.

**Methods:**

This study was conducted with 327 individuals aged 18-72 years, living in the Vale do Taquari area, Brazil. We assessed sociodemographic variables with a standardized protocol, symptoms of depression, anxiety, and stress with the Depression, Anxiety and Stress Scale-21 (DASS-21), sleep quality with the Pittsburgh Sleep Quality Index (PSQI), and post-traumatic stress symptoms with the Impact of Event Scale (IES-R), using a web-based online survey.

**Results:**

Our results showed that sleep dysfunction moderated the effects of age on psychological symptoms, indicating that younger participants who had poorer sleep quality had worse mental health. Furthermore, participants with more perceived stress during the pandemic and more sleep dysfunction reported more symptoms of anxiety and post-traumatic stress.

**Conclusion:**

Psychological symptoms were not related to social isolation duration but were related to the subjective perception that the pandemic interfered with life and generated stressful situations. These results may help governments make important decisions about protection and isolation measures in future waves of COVID-19 infection.

## Introduction

In December 2019, several pneumonia cases caused by a severe acute respiratory syndrome which was later diagnosed as COVID-19 were reported in Wuhan, China, resulting in a worldwide pandemic.^1^ In Brazil, the first case of COVID-19 was reported on February 26, 2020. Due to its complex epidemiological scenario, the country has been severely affected, with 231,534 deaths by the 8th of February, being considered the second most affected nation in the world.^2^ This scenario suggests that the responses from the relevant authorities could have been faster and more assertive.

Even when cases started to spike in non-urban areas, the federal government did not support social distancing measures, minimizing the pandemic’s importance.^3,4^ Therefore, with no national guidelines for prevention of the spread of the virus, decisions were made locally by state and district governments. On March 19, 2020, a state of public calamity was declared in the South of Brazil. As in many countries, this region adopted social distancing as a public health intervention, restricting movement and closing schools and businesses. However, such measures can also result in physical distancing of people who could provide social support from individuals who need it and, on an individual economic level, the possibility of an impending economic crisis and recession may result in many people losing their jobs and experiencing financial difficulties.^5^ Together with the fear of contracting the infection itself, these lifestyle changes have probably generated profound levels of anxiety.^6^ Moreover, some studies also suggest that another possible impact of the virus may be to worsen psychiatric conditions and neurological symptoms and that affected patients may be at higher risk of cognitive impairment after overcoming the COVID-19 infection.^7^

Several studies have reported the effects of the COVID-19 pandemic on the general population’s psychological outcomes in different countries.^8,9^ The main findings provide evidence of the negative impact of the COVID-19 pandemic on mental health in different populations. High rates of symptoms of anxiety, depression, anger, post-traumatic stress disorder (PTSD), and stress, and feelings of horror and apprehension were reported in the general population, especially in younger people, during the COVID-19 pandemic.^10,11^ Further, those with preexisting psychiatric disorders reported worsening psychiatric symptoms.^12^

Some studies have also reported worsening of quality of sleep in the general population, with an impact of a delay in Bed Time and Wake-Up affecting students in particular.^13^ This could be related to the increased risk of mental suffering for young people during the pandemic, since patterns of sleep disturbances have been consistently related to mental health and psychiatric disorders.^14^ Recent meta-analyses have also investigated the prevalence of sleep problems during the COVID-19 pandemic and found high rates of sleep disturbances in the general population (15% - 35.9%), in populations from several different countries around the world.^15-17^ However, findings relating to the pandemic’s impact on the population’s mental health have been considerably heterogeneous,^10^ hence the need for further studies.

Previous studies have reported findings on the COVID-19 pandemic and its impacts on mental health in the Brazilian population. Two studies included participants from all over the country, and one study included participants from one specific state.^9,18,19^ These studies found different results, identifying different social determinants as contributing to greater vulnerability to mental illness in the populations studied. One possible reason for this variability may be that these studies reported results from heterogeneous populations subjected to varying social distancing policies and did not focus on a single local profile subject to heterogenous prevention policies. Brazil is a country of continental dimensions, with significant differences between regions, especially in terms of social behavior, genetics, and economic backgrounds. Due to these marked regional differences, each of the country’s regions is at a different stage of the pandemic^20^ and mortality is exacerbated by regional disparities that exist within the health system.^21^ It is therefore important to evaluate the pandemic’s effects on mental health in a specific region in this country. Another important point is that none of these studies evaluated sleep quality using a specific scale. Considering that sleep quality has an important effect on mental health, it would be important to investigate its effects in this sample.

Thus, the purpose of the present study was to evaluate the impact of the COVID-19 pandemic on mental health in a single region in the South of Brazil, specifically the Vale do Taquari region. The region’s populations have similar culture and its local governments have adopted similar social distancing policies. More specifically, we first described the study population’s characteristics and their levels of stress, anxiety, depression, and sleep quality. Then, we wanted to see whether variables previously described as risk factors in the recent literature were also related to increased psychological distress, such as age, gender, education, and history of mental illness. Also, we investigated whether duration of social isolation and experiencing stressful situations during the pandemic led to increased psychiatric symptoms. Finally, we tested whether sleep quality partly explained the mental distress observed in the participants.

## Material and methods

This is a cross-sectional quantitative exploratory study conducted with individuals aged 18-72 years living in the Brazilian state of Rio Grande do Sul in a region called the Vale do Taquari area, which encompasses 36 towns and has approximately 351,999 habitants.^22^ The decision to use data from the Vale do Taquari was because this population has very high rates of COVID-19, ranking first in the state in terms of case count in May 2020,^23,24^ and also because of the homogeneity of the containment measures adopted to reduce spread of the virus in this region.

The subjects for this study were drawn from an online questionnaire conducted between August and September 2020. The questionnaire was constructed using the Google Forms platform. The questionnaire weblink was publicized on social networks, generating a snowball sample, in which participants shared the online questionnaire with their contacts. Only individuals who declared they resided in Vale do Taquari were considered for the analysis.

Electronic informed consent was obtained from all participants in accordance with the Declaration of Helsinki and the local ethics committee approved the study protocol.

Clinical and sociodemographic variables were assessed using a standardized protocol. The variable “stressful situations experienced during the pandemic” was measured as a yes/no answer to the following question: “Do you consider that you experienced stressful situations during the pandemic? (e.g., job loss, COVID-19 infection, bereavement or others).”

The severity of stress, anxiety, and depression, and sleep quality were measured with self-report assessment scales, as follows: the Depression, Anxiety and Stress Scale-21 (DASS-21)^25^ assesses the emotional states of depression (scores equal to or greater than 14 indicate moderate to severe depression), anxiety (scores equal to or greater than 10 indicate moderate to severe anxiety), and stress (scores equal to or greater than 19 indicate moderate to severe stress), with lower scores indicating less stressful impacts; the Impact of Event Scale-Revised (IES-R)^26^ is used to assess post-traumatic stress symptoms, with lower scores indicating a less stressful impact (a total score greater than 5.6 indicates psychological stress); the Pittsburgh Sleep Quality Index (PSQI)^27^ appraises sleep quality, using 19 items in seven dimensions that are scored from 0-3, and the total score, which was the sum of the scores from each dimension, ranges from 0-21 (scores equal to or greater than 5 indicate a disturbance in sleep quality), with higher scores indicating lower sleep quality. These instruments were not validated or adapted for online assessment. However, similar studies utilized the same scales in an online version.^7,10^

Descriptive statistics (number and %) were used to present data on sociodemographic characteristics, chronic medical diseases, and previous psychiatric disorders. Days of social distancing, age, and IES-R, PSQI, and DASS-21 scores were reported as mean and standard deviation. We used Pearson correlations to identify potential correlations between sex, age, household income, days of social distancing, previous psychiatric disorders, and education level and depression, anxiety, stress, and sleep quality. Finally, we conducted linear regression models to investigate the effects of age, sex, years of education, previous mental illness diagnosis, stressful situations experienced during the pandemic, social distancing duration, and sleep dysfunction on symptoms of depression anxiety, and stress. We estimated additional models with the following interactions individually to explore the possible mechanisms of psychological symptoms: sleep dysfunction with age; sleep dysfunction with a previous diagnosis of mental illness; sleep dysfunction with social distancing duration; and sleep dysfunction with experience of stressful situations during the pandemic. Analyses were conducted in SPSS version 18 and R version 4.0. Statistical significance was set at p < 0.05, and all tests were two-tailed.

## Results

A total of 327 individuals from 20 cities in the Vale do Taquari completed the survey. 244 (74.4%) of them were female, and their mean (SD) age was 36.46 (15.11) years. Of the total sample of respondents, 169 (51.7%) had a university degree or higher qualification, 284 (87.1%) were employed, 163 (49.8%) were married, 70 (21.4%) had a chronic disease, 34 (10.4%) reported a previous psychiatric disorder, 194 (59.3%) had experienced stressful situations during the pandemic, and 34 (10.4%) had had a confirmed diagnosis of COVID-19. The mean (SD) number of days in social distancing was 91.18 (55.49) and the mean (SD) household income was R$ 2,093.28 (10,202.70) (local currency- Brazilian reais).

The overall mean DASS-21 depression score for the participants was 6.74±6.18, reflecting a mild stressful impact, and 41.6% of respondents reported moderate to severe depressive symptoms. The mean DASS-21 anxiety score was 5.36±5.45 (moderate), and 45.9% of respondents reported moderate to severe anxiety symptoms. The mean DASS-21 stress score was 9.16±6.02 (mild), and 44.6% reported moderate to severe stress levels. The overall mean IES-R total score was 3.44±3.14 and 94 (28.74%) subjects reported PTSD symptoms. The mean PSQI score for sleep quality was 7.31±3.91, indicating significant sleep disturbance, and 73.5% of respondents reported disturbance in sleep quality.

Table 1 shows the results for correlations between PTSD, symptoms of stress, depression, and anxiety, and sleep quality and respondents’ clinical features. Age and years of education were negatively correlated with PTSD, with symptoms of stress, depression, and anxiety, and with sleep quality (all p < 0.05). Gender was not related to any of the other variables. Income was negatively correlated with stress symptoms, and social distancing was positively correlated with PTSD symptoms (all p < 0.05). Previous psychiatric disorders were correlated with PTSD, with symptoms of stress, depression, and anxiety, and with poor sleep quality (p < 0.05).

**Table 1:** Correlations between PTSD, sleep quality, symptoms of stress, depression and anxiety, sex, age, income, social distancing, psychiatric disorders, and educational level

Variable	IES-R			DASS-21 depression			DASS-21 anxiety			DASS-21 stress			PSQI		
	N	R	p	N	R	p	N	R	p	N	R	p	N	R	p
Age	327	-0.371	**< 0.001**	327	-0.456	**< 0.001**	327	-0.413	**< 0.001**	327	-0.525	**< 0.001**	310	-0.137	**0.016**
Income	305	-0.100	0.080	305	-0.106	0.065	305	-0.096	0.094	305	-0.129	**0.024**	288	-0.004	0.949
Social distancing	325	0.122	**0.028**	325	0.087	0.117	325	0.076	0.173	325	0.074	0.181	308	0.091	0.111
Years of education	327	-0.158	**0.004**	327	-0.259	**< 0.001**	327	-0.184	**0.001**	327	-0.168	**0.002**	310	-0.150	**0.008**
Gender	326	-0.071	0.203	326	-0.061	0.272	326	-0.115	0.038	326	-0.097	0.081	309	-0.029	0.617
Psychiatric disorders	327	0.204	**< 0.001**	327	0.212	**< 0.001**	327	0.150	**0.006**	327	0.181	**0.001**	310	0.169	**0.003**

DASS-21 = Depression, Anxiety and Stress Scale-21; IES-R = Impact of Event Scale-Revised; PSQI = the Pittsburgh Sleep Quality Index.

**Figure 1:**
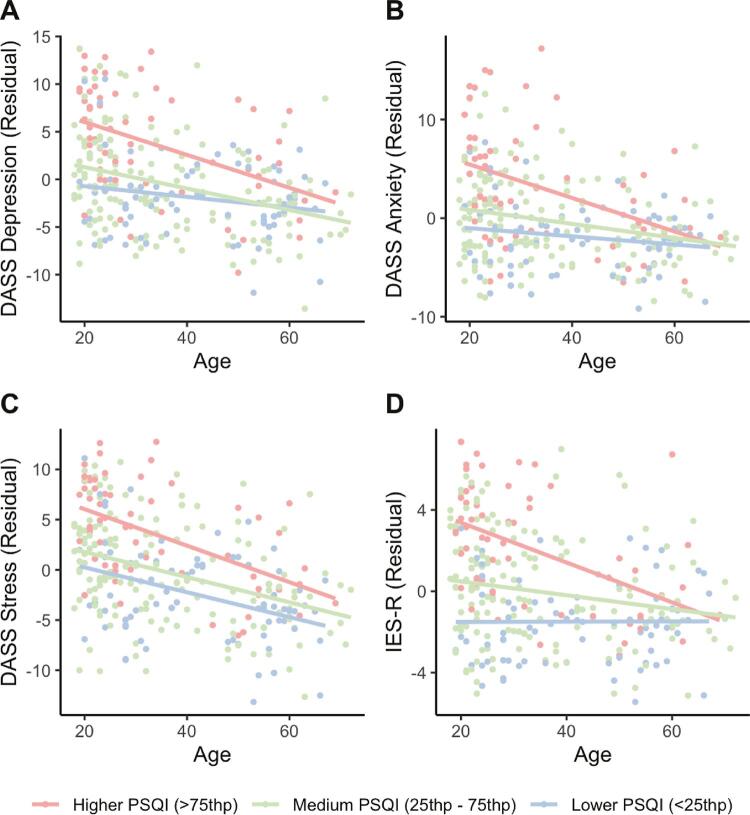
Age by sleep interactions predicting psychiatric symptoms, controlling for gender, years of study, previous psychiatric diagnosis, duration of social isolation, and perceived stress during the pandemic.

A model predicting the DASS depression subscale in terms of age, gender, education, history of mental illness, social isolation duration, stressful situations experienced during the pandemic, and PSQI (F_7,297_ = 31.97, p < 0.001, Adj. R^2^ = 0.42) indicated that depressive symptoms were related to age (t = -7.156, p < 0.001, b = -0.33), education (t = -3.374, p < 0.001, b = -0.15), stressful situations experienced during the pandemic (t = 4.061, p < 0.001, b = 0.19), and sleep dysfunction (t = 7.099, p < 0.001, b = 0.33). In models including the previous variables as dependent variables and additional interaction terms, we found an interaction between age and sleep dysfunction (t = -2.214, p = 0.03, b = -0.30; model: F_8,296_ = 28.95, p < 0.001, Adj. R^2^ = 0.42), indicating that sleep dysfunction was more important to depressive outcomes for younger participants than for older ones (Figure 1A). We did not find any interactions between sleep dysfunction and history of mental illness (t = 0.823, p = 0.41, b = 0.09; model: F_8,296_ = 28.03, p < 0.001, Adj. R^2^ = 0.42), social distancing duration (t = -0.733, p = 0.46, b = -0.09; model: F_8,296_ = 28.0, p < 0.001, Adj. R^2^ = 0.42), or stressful situations experienced during the pandemic (t = 1.582, p = 0.11, b = 0.20; model: F_8,296_ = 28.43, p < 0.001, Adj. R^2^ = 0.42).

Lower PSQI (n = 82), Medium PSQI (n = 165), Higher PSQI (n = 63).

A model predicting the DASS anxiety subscale scores in terms of age, gender, education, history of mental illness, social isolation duration, stressful situations experienced during the pandemic, and PSQI (F_7,297_ = 31.97, p < 0.001, Adj. R^2^ = 0.37) indicated that anxiety symptoms were related to age (t = -6.101, p < 0.001, b = -0.29), stressful situations experienced during the pandemic (t = 3.646, p < 0.001, b = 0.18), and sleep dysfunction (t = 7.328, p < 00.001, b = 0.36). In further models with the previous variables as dependent variables and additional interaction terms, we found an interaction between age and sleep dysfunction (t = -3.345, p < 0.001, b = -0.47; model: F_8,296_ = 25.03, p < 0.001, Adj. R^2^ = 0.39), indicating that sleep dysfunction was more important to anxiety outcomes for younger participants than for older ones (Figure 1B). Also, we found an interaction between a previous mental illness diagnosis and sleep dysfunction (t = -2.112, p = 0.04, b = -0.26; model: F_8,296_ = 23.67, p < 0.001, Adj. R^2^ = 0.37), although this result would not have been upheld if controlling for multiple comparisons. We did not find an interaction between sleep dysfunction and social distancing duration (t = 0.341, p = 0.74, b = -0.05; model: F_8,296_ = 22.8, p < 0.001, Adj. R^2^ = 0.37), but we did find an interaction between stressful situations experienced during the pandemic and sleep dysfunction (t = 2.315, p = 0.02, b = 0.30; model: _F8,296_ = 23.86, p < 0.001, Adj. R^2^ = 0.38).

A model predicting DASS stress subscale scores in terms of age, gender, education, history of mental illness, social isolation duration, stressful situations experienced during the pandemic, and PSQI (F_7,297_ = 40.07, p < 0.001, Adj. R^2^ = 0.47) indicated that stressful symptoms were related to age (t = -9.383, p < 0.001, b = -0.41), stressful situations experienced during the pandemic (t = 3.955, p < 0.001, b = 0.18), and sleep quality (t = 8.076, p < 0.001, b = 0.36). In further models with the previous variables as dependent variables and additional interaction terms, we did not find any interactions between sleep dysfunction and age (t = -1.106, p = 0.27, b = -0.15; model: F_8,296_ = 35.24, p < 0.001, Adj. R^2^ = 0.47, Figure 1C), previous mental illness diagnosis (t = -1.263, p = 0.21, b = -0.14; model: F_8,296_ = 35.33, p < 0.001, Adj. R^2^ = 0.47), social distancing duration (t = -1.454, p = 0.15, b = -0.18; model: F_8,296_ = 35.46, p < 0.001, Adj. R^2^ = 0.48), or stressful situations experienced during the pandemic (t = 1.401, p = 0.16, b = 0.17; model: F_8,296_ = 35.42, p < 0.001, Adj. R^2^ = 0.48).

A model predicting IES-R scores in terms of age, gender, education, history of mental illness, social isolation duration, stressful situations experienced during the pandemic, and PSQI (F7,297 = 28.57, p < 0.001, Adj. R^2^ = 0.39) indicated that PTSD risk was related to age (t = -5.388, p < 0.001, b = -0.25), stressful situations experienced during the pandemic (t = 3.202, p = 0.0002, b = 0.15), and sleep dysfunction (t = 8.738, p < 0.001, b = 0.42). There was a significant main effect for social distancing duration (t = 2.030, p = 0.04, b = 0.09), although this result would not have been upheld if controlling for multiple comparisons. In further models with the previous variables as dependent variables and additional interaction terms, we found an interaction between age and sleep dysfunction (t = -3.451, p < 0.001, b = -0.48; model: F_8,296_ = 27.41, p < 0.001, Adj. R^2^ = 0.41), indicating that sleep dysfunction was more important to post-traumatic stress symptoms for younger participants than for older ones (Figure 1D). Also, we found an interaction between previous mental illness diagnosis and sleep dysfunction (t = -2.023, p = 0.04, b = -0.24; model: F_8,296_ = 25.77, p < 0.001, Adj. R^2^ = 0.39), although this result would not have been upheld if controlling for multiple comparisons. We did not find any interaction between sleep dysfunction and social distancing duration (t = -0.796, p = 0.43, b = -0.11; model: F_8,296_ = 25.05, p < 0.001, Adj. R^2^ = 0.39), but we did find an interaction between stressful situations experienced during the pandemic and sleep dysfunction (t = 2.355, p = 0.02, b = 0.30; model: F_8,296_ = 26.07, p < 0.001, Adj. R^2^ = 0.40).

## Discussion

Our study investigated the impact of the COVID-19 pandemic on the general public’s mental health and sleep quality in the South of Brazil. We included a sample from a specific area where the public health interventions implemented are similar, to control for possible confounders of the pandemic’s consequences. Our results showed that a significant proportion of participants reported moderate to severe symptoms of anxiety, depression, stress, and post-traumatic stress, which were mainly related to age, to experiencing stressful situations during the pandemic, and to sleep dysfunction, but not to female gender or previous mental illness diagnosis. More importantly, sleep dysfunction moderated the effects of age on psychological symptoms, indicating that younger participants who had lower sleep quality reported worse mental health. Further, participants who experienced stressful situations during the pandemic and had more sleep dysfunction reported more symptoms of anxiety and post-traumatic stress. Finally, psychological symptoms were not related to duration of social isolation per se, but to the subjective perception that the pandemic interfered with life and generated stressful situations. As far as we know, our study is the first to describe these alarming relationships in the context of the COVID-19 pandemic. These findings are further discussed below.

Our results demonstrated that more than forty percent of the participants scored moderate to severe for symptoms of anxiety, depression, and stress. Moreover, 28.74% of the subjects reported post-traumatic stress symptoms, while 59.3% considered they had experienced stressful situations during the pandemic. When compared to another study^9^ that investigated the prevalence of psychiatric symptoms among the general Brazilian population during the peak of the pandemic, our rates of symptoms of depression and anxiety are lower. However, our finding is worrying because these percentages are higher when compared to other studies,^28-32^ but are consistent with findings from a recent meta-analysis.^33^ One possible reason for these differences may be the homogeneity of our sample, reflecting the consequences of the pandemic in a specific context, with similar adherence to social distancing recommendations. Additionally, when our study was conducted, this region was suffering from high rates of COVID-19, with new cases and deaths. By September 2020, the number of confirmed COVID-19 cases in Vale do Taquari was approximately 9,000; in other words, 2.7% of the population.^23^ Another critical aspect to consider is that this region has high suicide rates.^34^ It is well established that the majority of suicides and suicide behavior worldwide are related to psychiatric diseases and psychiatric symptoms.^35^

However, contrary to previous findings,^12,36^ a previous diagnosis of mental illness was not a main predictor of psychological symptoms when controlling for other variables. This might indicate an impact from the pandemic on the participants’ mental health and not just an exacerbation of psychiatric disorders during this period. Along the same lines, a study by Dawel et al.^37^ that evaluated a sample from the Australian population in the early acute phase of the COVID-19 pandemic found that depression and anxiety symptoms were substantially elevated even in individuals with no existing mental health diagnoses. Interestingly, our results showed that psychological symptoms were related to the subjective perception that the pandemic interfered with life and generated stressful situations such as financial worry, fear of contracting the infection, and concerns for loved ones’ health. A recent study indicated that the pandemic’s perceived negative impact on livelihood showed a large effect size for predicting mental health problems.^38^ Conversely, psychological symptoms were not related to social isolation duration in our sample, indicating that the mental distress might be attributable to fear of the pandemic and not due to the governments’ restrictive measures. Consistent with our data, a recent study showed that the effects of mitigation measures, such as stay-at-home orders, did not involve increases in more severe psychopathology, such as clinical depression, or suicide ideation/planning in the general population, revealing that psychological distress related to social isolation is likely to be low compared to the health benefits of mitigating the COVID-19 pandemic.^39^

We also did not find a significant effect of gender in predicting mental health outcomes, contrary to the high levels of psychological distress among women found in a previous study,^10^ but consistent with a recent meta-analysis.^28^ However, younger age was associated with anxiety, depression, stress, and PTSD levels, which is supported by the literature.^11,40^ Being younger and following COVID-19 news updates seems to be associated with elevated levels of anxiety compared with those who were older and those who were less exposed to COVID-19 news updates,^11^ suggesting that young people might be more vulnerable to the impacts of their surroundings.

Interestingly, sleep dysfunction moderated the effects of age on psychological symptoms, indicating that younger participants who had lower sleep quality had worse mental health. This result is consistent with the literature, which shows that anxiety was associated with stress and reduced sleep quality in a sample from China,^41^ and that younger people had increased risk of developing sleep disturbances as well as higher levels of anxiety and distress in a sample from Italy.^6^ There are reports showing disturbances in sleep quality and sleep habits in a significant proportion of the population during the pandemic.^42^ Reduced sleep quality negatively affects functioning and is associated with a burden of comorbid psychiatric symptoms in young people.^43^ Another important factor possibly interfering in this effect is the increased social communication between people and the time spent in front of cell phones and computers. This kind of interaction is not the same as face-to-face social interaction, and the long screen exposure time may affect sleep quality, mainly when use is close to bedtime.^44^ As stated by other authors,^45^ during home confinement, people reported increased time on social media soon before bedtime, more time spent in bed, and lower sleep quality compared to the pre-lockdown period, and this pattern was more pronounced in those with higher levels of depression, anxiety, and stress symptomatology. Futures studies should investigate whether increased use of social media, internet use, and news consumption are associated with sleep disturbance in young participants.

Moreover, sleep quality also interacted with experiencing stressful situations during the pandemic in prediction of symptoms of anxiety and post-traumatic stress. It is well known that concerns about one’s own health or relatives’ health, experiencing changes to routines, living with uncertainty and with stress, and worries about the current situation may influence sleep quality and generate or exacerbate fear, depression, and anxiety.^44,46^ Thus, poor sleep quality seems to be an important marker of worse mental health during the pandemic. Sleep can be thought of as a transdiagnostic trait linked to different mental disorders^47^ and its quality is associated with emotional regulation. Healthy sleep may be protective for dealing with challenges,^44^ although a previous report indicates different sleep quality patterns during the pandemic, partially influenced by pre-pandemic sleep characteristics.^48^

Some limitations of this study must be mentioned. First, we had a modest sample size compared to previous studies.^9,32^ Second, this was a web-based study, so only participants with internet access could respond to the questions, not allowing universal access. Third, we did not include a pre-pandemic comparison group and although symptoms of depression and anxiety are moderate to severe during the pandemic, it is difficult to state whether this prevalence is equal to or greater than before the pandemic. Fourth, we included only scales that are not validated for online assessment. Fifth, there is a possibility of selection bias, because there is a chance that only individuals who were struggling with their mental health during the pandemic would be interested in answering the questionnaire. This bias mostly impacts the prevalence reported by the study. Sixth, there are no available epidemiological data from Vale do Taquari that allow us to make detailed comparisons with our sample. Seventh, the analyses were not controlled for multiple comparisons. However, all linear regression model p-values were < 0.001, thus they would have been maintained with any method. Eight, the cross-sectional design does not enable evaluation of causality. Future studies with a follow-up design will help clarify the consequences of the COVID-19 pandemic in the mental health population. It would also be interesting to investigate whether the participants develop post-traumatic stress symptoms after the COVID-19 pandemic is over. Finally, all outcomes were self-reported rather than evaluated by a clinician.

Nonetheless, this report is noteworthy in several respects. This study was conducted in a sample from a specific region where the culture and strategies to deal with the pandemic are similar, prioritizing the specific local characteristics. Our main finding showed an impact on mental health and sleep quality, especially in younger people during the COVID-19 pandemic. However, these consequences seem not to be associated with isolation per se, but with experiencing stressful situations during the pandemic. These results may help the federal or local governments make important decisions about protection and social-distancing measures in subsequent waves of COVID-19 infection. These results may also help healthcare workers manage the consequences of the coronavirus outbreak for the population’s mental health, developing new strategies to improve sleep quality. Focusing on the general population’s sleep quality could mitigate the impact of the pandemic on mental health, especially in young people. Limiting sources of stress, maintaining the usual sunlight exposure, physical activity, and sleep-wake rhythms pattern, and focusing on the benefits of isolation (saving our own lives and protecting others from the virus),^46^ may help to deal with the adverse consequences of this period. Further, non-pharmacological interventions such as light/dark exposure and regulation of biological and behavioral rhythms could yield significant benefits for mental health during the COVID-19 pandemic.^47^ Likewise, the relevant authorities should be aware of the pandemic’s possible psychological impacts and establish national guidelines to promote mental health to minimize subjective feeling of stress and poor sleep quality during the COVID-19 pandemic.
